# Polishing systems for modern aesthetic dental materials: a narrative review

**DOI:** 10.1038/s41415-024-7963-x

**Published:** 2024-10-25

**Authors:** Adil Khan, Nicholas Hodson, Asmaa Altaie

**Affiliations:** 41415162927001 Clinical Teaching Fellow in Restorative Dentistry, Level 6 Worsley Building, Leeds Dental Institute, Clarendon Way, Leeds, LS2 9LU, UK; 41415162927002 Professor/Honorary Consultant in Restorative Dentistry, Level 6 Worsley Building, Leeds Dental Institute, Clarendon Way, Leeds, LS2 9LU, UK; 41415162927003 Senior Clinical Lecturer/Honorary Consultant in Restorative Dentistry, Level 6 Worsley Building, Leeds Dental Institute, Clarendon Way, Leeds, LS2 9LU, UK

## Abstract

**Objectives** To review the current literature surrounding chairside polishing systems for resin composites, zirconia and lithium disilicate restorations.

**Methods** A literature search was undertaken and databases were hand-searched for the most relevant articles.

**Discussion** The current marketplace contains a wide variety of polishing systems, each with different abrasive compounds and number of steps. Current efforts are aimed at reducing the number of steps required for polishing to improve clinical effectiveness. Reduced step systems showed some comparable results to the more traditional multi-step protocols, but the most effective results were achieved with the use of polishing paste as an additional step.

**Conclusions** Based on the current available literature, the use of material-specific polishing systems is effective for chairside polishing of direct and indirect restorative materials. However, it is important to emphasise that, for optimum outcomes, it is essential to follow manufacturers' recommendations for each step, with particular considerations of the handpiece speed, time spent per step and use of adjunct water coolant.

## Key points

- Provides information on the importance of polishing and finishing direct and indirect restorations.
- Gives dental practitioners an overview of the various systems that are available.
- Proposes a decision-making flowchart for practitioners to use in choosing an appropriate system to use.
- Suggests an appropriate protocol to use for commonly used aesthetic materials.

## Introduction

Having been around since the 1950s, dental composites are by no means a recent development, but they have undergone significant changes over this period of time.^1^^,^^2^ This is mainly directed at the filler dimensions and concentrations, but there has been some alteration to the monomer components.^3^^,^^4^ There are now a variety of composites available, including nanofills, microhybrids, ‘bulk-fill' and flowables, among others.^1^^,^^2^^,^^3^ The variations in the flow and deformation of these materials coupled with aesthetics have now led to composites being the typical direct restoration of choice, especially with the continued attempt to phase out amalgam following the Minamata Convention.^4^^,^^5^

With regard to indirect restorations, feldspathic ceramics and metal alloy substructures were developed in the 1960s and have been widely used since.^1^^,^^6^ These materials and subsequent developments relied on layered restorations which were prone to chipping.^6^ Therefore, there has been a recent drive to develop restorations in a monolithic form to avoid this.^1^ Lithium disilicate was developed in the late 1990s and is very commonly used in practice for indirect restorations.^6^^,^^7^ Initially offered as part of a layered system, advances in manufacturing meant it could be delivered in a monolithic form.^7^^,^^8^ Lithium disilicate offers good survival rates in various applications but only in the short- to medium-term as there is not enough research to assess their long-term survival rate.^6^^,^^7^^,^^8^ Zirconia was developed around a similar time and comes in many forms, but the main form used in dentistry is the partially yttria-stabilised tetragonal phase.^8^^,^^9^^,^^10^ Again, it was initially used as part of a layered restoration, but due to the issue of chipping, there has been a recent drive fabricating full-contour zirconia restorations.^11^ Monolithic zirconia restorations have been shown to be particularly robust due to their extremely high mechanical properties, as well as being biocompatible.^6^^,^^10^^,^^11^ However, the main issue has been achieving optimum aesthetics as they are typically quite opaque in nature.^8^^,^^12^ Manufacturing methods have attempted to overcome this, using techniques such as adding coloured pigments and introducing translucent versions, but it is recommended to either use a layered restoration or a different material in highly aesthetic areas.^8^^,^^12^

Finishing and polishing is a vital step in the placement of any direct restoration or following adjustment of any indirect restoration. Furthermore, these steps have led to other benefits, including reduced risk of fracture; reduced surface imperfections which would reduce plaque accumulation and surface breakdown; reduced wear on opposing and adjacent teeth; and production of a more aesthetic, light-reflective surface.^2^^,^^6^^,^^11^^,^^13^^,^^14^^,^^15^ Reduced surface roughness and increased surface gloss is an important part in achieving an aesthetic optimum outcome. Additionally, there are functional benefits, as a smoother surface will reduce the risk of crack formation and propagation that lessen the overall risk of fracture.^13^ Finishing relates to the contouring of the restoration to achieve the desired anatomy, as well as any occlusal adjustments, whereas polishing is specifically the use of instruments to reduce the surface irregularities created during the finishing process.^15^ There are a number of polishing systems available, from multi-step protocols to more simple one- and two-step systems, which were recently introduced in an attempt to save chairside clinical time.^2^^,^^13^

The purpose of this review is to provide an analysis of the current chairside polishing systems available for these modern, aesthetic, direct and indirect restorative materials.

## Methods

An outline of the most commonly used aesthetic dental materials, both direct and indirect, was created. A subsequent literature search of articles related to the effectiveness of different polishing systems which used surface roughness or surface gloss as one of their parameters. An initial abstract screen was conducted through databases (PubMed and Medline) looking for key words, including finishing and polishing, as well as any reference to the materials we were considering - direct composites, zirconia and lithium disilicate. Composite used as an indirect material was not considered here. Following the initial abstract screen, a full-text review of potentially relevant articles was undertaken. Articles must have measured either surface roughness or surface gloss as part of the study, with the raw scores available within the article allowing a comparison between different papers. The protocols used for finishing and polishing must have been detailed clearly, with at least the brand name mentioned. Other information, such as polishing grit and number of steps within the protocol, was desirable but not essential as this could be researched using the brand name on the manufacturer's website. Proceeding this, a narrative description of the key results was used in the main text, with the main body separated into different segments according to each material.

## Results and discussion

### Resin composite restorations

**Fig. 1:**
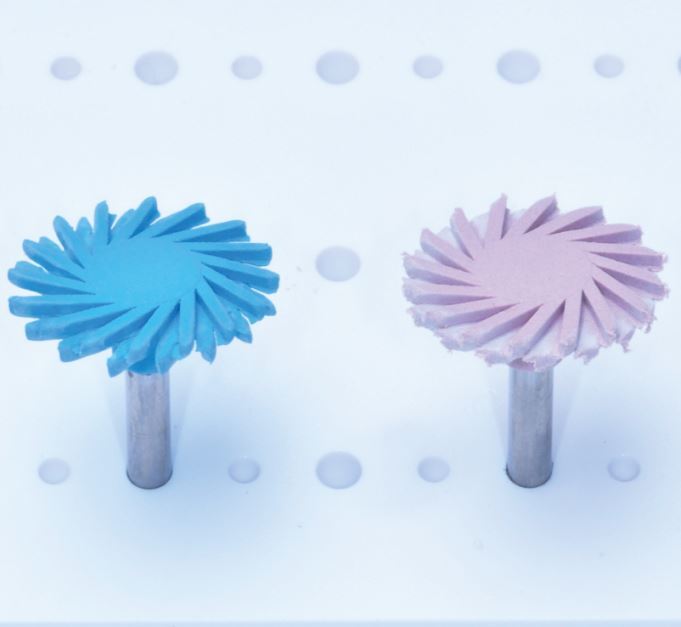
DIATECH ShapeGuard Composite Plus Polishing Kit by Coltene - a two-step system

**Fig. 2:**
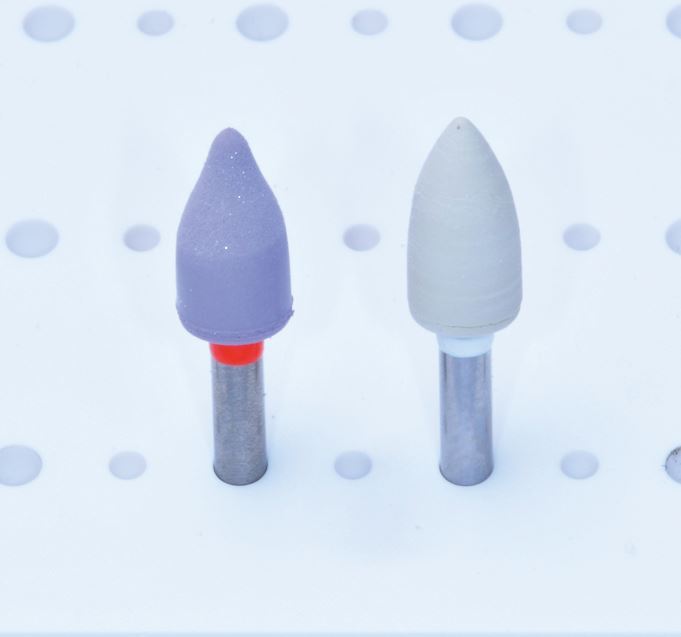
Complete Polishing System for Composite by Kenda - a two-step system

**Fig. 3:**
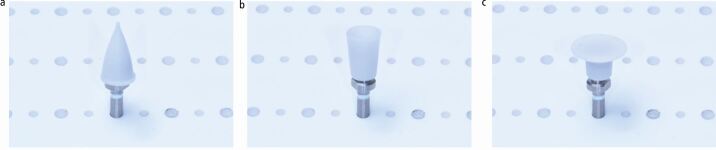
a, b, c) OneGloss Polishing System by Shofu - a one-step system with point, cup and disk forms

Polishing is not the only variable that affects the final roughness and surface gloss of the restoration; it is important to note that the filler particle size used in the material is also an important factor.^16^^,^^17^ For a quality polishing system, it is generally accepted that a roughness below 0.2 μm is the threshold as above this, bacteria retention can occur and patients can detect roughness above 0.3 μm with their tongue.^18^^,^^19^ Using Mylar strips alone can result in the smoothest surfaces;^16^ however, this produces an outer layer that is rich in resin which is prone to degradation and requires removal through polishing to produce a more functional and aesthetic restoration.^20^^,^^21^ Various compounds are used in polishing protocols, such as aluminium oxide, silicon dioxide and diamond abrasives.^13^ Some of the tools used include rubber cups and points, abrasive polishing discs and dental stones. Diamond and carbide burs are used in the finishing process but these are typically for initial gross reduction and leave a rough surface that requires further polishing.^13^ A summary of some different polishing systems available is shown in Table 1 (see Fig. 1, Fig. 2 and Fig. 3).^22^

**Table 1:** Table indicating the different composite polishing systems available

**Steps**	**Composition**	**Brand name (abrasive)**	**Manufacturer**
Multi-steps	Abrasive discs	Super-Snap − (SC/AO)	Shofu
		Soflex Discs − (AO)	3M
		Maxflex Pop-On − (AO)	Stoddard
		OptiDisc − (AO)	Kerr
		SeptoDiscs − (AO)	Septodont
		EVE Flexi-D − (AO)	Eagle Dental
		EP Polishing System − (?)	Brasseler
		Jiffy Spin − (D)	Ultradent
		FlexiDisc − (AO)	Cosmedent
		VersaFlex − (D)	Brasseler
	Rubber points/cups/discs	Astropol − (AO)	Ivoclar Vivadent
		Identoflex Composite − (D)	Kerr
		Jiffy Polishers − (D)	Ultradent
		Set 4312A − (D)	Komet
Two steps	Rubber points/cups/discs	Composite − (A)	Shofu
		Venus Supra (D)	Kulzer
		HiLuster − (D)	Kerr
		Diagloss − (D)	Edenta
		DIATECH ShapeGuard Composite Plus − (D)	Coltene (Fig. 1)
		Diacomp − (D)	Brasseler
		Twist Dia for Composite − (D)	Kuraray
		FlexiCups/FlexiPoints − (AO)	Cosmedent
		Art2 Polishers − (D)	Komet
		Complete Polishing System for Composite − (D)	Kenda (Fig. 2)
Single steps	Rubber points/cups/discs	Enhance PoGo − (D)	Dentsply Sirona
		OptraGloss (D)	Ivoclar Vivadent
		Opti1Step − (D)	Kerr
		TopGloss (D)	Edenta
		OneGloss − (A)	Shofu (Fig. 3)
		ComposiPro One-Step − (D)	Brasseler
		Jiffy One − (D)	Ultradent
		SeptoPolisher − (AO)	Septodont
		9523/9525 One-Step Composite Polishers − (D)	Komet
	Brushes	Occlubrush − (SC)	Kerr
		ComposiPro Brush − (SC)	Brasseler
	Polishing stones	Dura-White − (AO)	Shofu
Adjuncts	Polishing paste	Diamond − (D)	Various
		Aluminium oxide − (AO)	Various
	Post-polish brushes	Jiffy Brush − (SC)	Ultradent
Key: A = Alumina AO = Aluminium oxide D = Diamond SC = Silicone carbide ? = Unknown

Within the current published research, there are conflicting views as to the most effective system to use. Previously, it was accepted and shown that the multi-step abrasive discs were clinicians‘ choice of system.^16^^,^^23^ However, with newer products coming out aimed at increasing convenience and time efficiency, the reduced-step polishing systems have shown equal if not better results to the traditional systems, as well as achieving surface roughness below 0.2 μm.^17^^,^^21^^,^^24^^,^^25^ This can lead to confusion when evaluating which polishing system to use as it is impractical to have multiple systems in the clinical setting.

Recent systematic reviews in the area do indicate that the multi-step abrasive discs are the system with the highest probability of achieving the lowest surface roughness.^22^ The main systems mentioned in these reviews were the Sof-Lex and Super-Snap systems.^26^ One review did highlight that the best mean surface roughness was achieved by the Astropol system.^26^ The common denominator featured is the use of aluminium oxide within the polishing system which is harder than the composite, allowing removal of both the matrix and filler components of composite.^22^^,^^26^ Diamond is also harder than composite, and systems containing diamond particles infused within them, or the use of adjuncts such as diamond polishing pastes, also achieved surface roughness below 0.2 μm.^13^

Systems that achieved the desired threshold for surface roughness varied between two and five steps, indicating that single-step systems are not as effective currently; although, the results are not far off and the PoGo and Enhance systems mentioned these could be a ‘moderately acceptable option' as they were close to the 0.2 μm threshold while simplifying the clinical workflow.^26^ Combining different systems was also a variable considered but it was not clear that this had a significant effect and is likely to be impractical in a clinical setting with its increased complexity and number of steps.^26^

There are various other factors to consider that can affect the outcome, such as type of composite used, correct application of the system according to the manufacturer's instructions (including time and force applied) and operator skill.^22^ In addition to this, these studies were all laboratory studies and the clinical scenarios faced by clinicians can vary.^22^^,^^26^

Whereas the single-step systems do not show optimal results currently, the diamond-based reduced-step systems have shown results that emulate the outcomes of the multi-step systems.^27^ These systems have been presented in isolated studies rather than in any reviews but they show promise and using them in clinical practice will be able to save chair time. Additionally, as some of the elastomer-based systems can be sterilised and reused, clinical waste could be significantly reduced.^24^

### Zirconia restorations

Even with advances in computer-aided design/computer-aided manufacturing (CAD/CAM) technology, it is common for indirect restorations to require adjustment after cementation.^28^ Grinding done with diamond burs can significantly increase the surface roughness of the restoration.^29^ Polishing is an important step to improve the aesthetics and performance of the restoration, as well as reduce the risk of wear of the opposing tooth.^28^ It has been reported that a suitable and effective polishing protocol can result in a smooth surface comparable to the initial glazed restoration and can be achieved regardless of experience.^30^^,^^31^

Initially, polishing was done with general ceramic polishing systems before the advent of zirconia-specific systems. Modern zirconia systems have been shown to have significant improvements in polishing capabilities compared to the previous generic ceramic systems.^32^ Zirconia polishing systems need to have particles which are harder than zirconia itself to be effective and therefore, the majority of them have diamond as their primary agent; although, there are alternatives, such as aluminium oxide or even zirconia itself.^33^^,^^34^

In comparison to composite finishing and polishing, zirconia systems need multiple steps (minimum of two) to achieve an acceptable outcome, with a focus on course-grit finishing and medium-grit polishing.^35^ Research assessing surface roughness outcomes showed that all zirconia systems result in significant improvement compared to grinding alone. However, when we take into consideration the 0.2 μm threshold, only certain systems reached this goal including Meisinger (LUSTER for Zirconia Adjusting and Polishing Kit), EVE (EVE Diacera), Cerapro (Cerapro StarGloss), Jota (Jota Kit 1434) and Edenta (Magic KIT Zirconia).^29^^,^^33^^,^^35^ Even other systems that did not reach the threshold still produced clinically acceptable results as the benchmark should be a similar roughness to ungrounded glazed zirconia. These included the Shofu (CeraMaster Assorted) and Brasseler (Dialite ZR) systems,which are both two-steps.^36^

Manufacturers usually have recommended revolutions per minute (RPMs) settings for each stage to achieve the required results. However, there is some evidence that indicates 15,000 RPM was the preferred speed to achieve low roughness and high gloss.^36^ In relation to the duration of polishing, manufacturers do not typically recommend a specific time for each polisher; however, it has been previously reported that 60 seconds with water coolant is the ideal marker to achieve the best result with each polisher, without affecting the structure of the restoration.^37^

An interesting adjunct to consider which can improve the overall polish is the additional use of diamond polishing paste after the chosen polishing system. Some studies have suggested the use of any diamond polishing paste can result in lower surface roughness and increased glossiness well within the desired thresholds.^34^^,^^38^ The main suggestion would be to use a specific zirconia polishing kit and to use the full range of the system chosen rather than missing out steps. Use of polishing paste as a final step would also be ideal, particularly if the system used is a two-step protocol which may not have a ‘super-fine' polisher. However, this is all based off *in vitro* studies which may not be representative of the clinical scenario and the lack of systematic reviews in this area needs to be addressed before coming to any strong recommendations.

### Lithium disilicate restorations

Similarly to zirconia, indirect restorations made of lithium disilicate can require adjustments following cementation.^28^ However, it is more important to address this, as unglazed surfaces wear at a much faster rate compared to glazed or polished surfaces.^39^ Furthermore, the lithium disilicate itself can wear away at a faster rate; therefore, it is important to polish the surface following any grinding.^40^

ISO 6872 (dentistry - ceramic materials) specifies the requirements for ceramic materials testing and it recommends pre-testing polishing as an essential step to minimise surface irregularities and surface flaws resulting in subsurface cracks and crack propagation, which negatively impact on the mechanical performance of the restorations.^41^

There are many different systems available to choose from which can make it difficult to pick and in a clinical setting, it may be tempting to use one system for various materials to save costs. However, it has been indicated that ceramic polishing systems produced significantly smoother surfaces compared to composite systems on lithium disilicate.^42^ On the other hand, some studies do indicate that some zirconia and multi-purpose ceramic polishing systems can obtain comparable results to lithium disilicate-specific polishing systems.^43^^,^^44^^,^^45^ This may indicate that a similar system can potentially be used for both zirconia and lithium disilicate but it must be noted that these are *in vitro* studies and a lack of reviews means that this cannot be adopted as a formal recommendation. This is evidenced by another study which found that a lithium disilicate polishing kit outperformed the zirconia polishing system on both lithium disilicate and zirconia.^46^

Within the available literature, numerous lithium disilicate polishing systems have been tested (Brasseler Dialite HP and LD systems, Ivoclar Vivodent OptraFine, Komet LD/ZR, Shofu Ceramisté, Diatech Cerashine, Wieland Zenostar, Edenta Ceropol). These vary between two and three steps and all have been shown to significantly reduce the surface roughness of roughened lithium disilicate.^47^^,^^48^ Despite improving the surface, none were able to reach the smoothness of the initial glazed surface. However, the OptraFine system incorporates a final step in their protocol which uses diamond polishing paste.^49^ When this was then used, the roughness was equivalent to the initial glazed surface.^47^ Other studies that incorporated the OptraFine system and used the whole protocol also found comparable results and that the system produced better results compared to other systems it was pitted against.^49^^,^^50^ Without this final step, the system was outperformed; therefore, a final step with diamond paste may be beneficial regardless of what system is used.^47^

The majority of studies are *in vitro* but a clinical study looked at two of the three-step systems, both augmented with an additional polishing step with diamond paste. The roughness achieved was comparable to the initial glazed restoration.^51^ Despite the follow-up period being short, there were no failures reported over the year and this can support the use of diamond paste regardless of what system is chosen.

Similarly to zirconia, to obtain the best outcome from the system chosen, it has been indicated that each step should be performed for 60 seconds to achieve the lowest roughness and the highest gloss with water coolant.^52^ Using it for less results in a poorer outcome, regardless of whether it is the rubber point or the polishing paste.^53^

In relation to the number of steps, one study indicated that two-step systems produced comparable results to three-step systems. However, the roughness values achieved were relatively high and this could be attributed to each step only being used for 10 seconds which is sub-optimal.^54^

An interesting, more recent development is the use of cerium oxide in the form of an alumina-ceria polishing paste. Cerium oxide can polish lithium disilicate via a chemo-mechanical process rather than just mechanical alone, which is what the current, widely used diamond pastes do.^55^ Current results indicate that it does have a superior effect when compared to diamond pastes and it has even been theorised that it could be used on its own, which would be beneficial in the clinical context.^56^ However, more studies are required and further comparisons made to the current systems on the market currently before any firm conclusions can be made.

## Conclusion

**Fig. 4:**
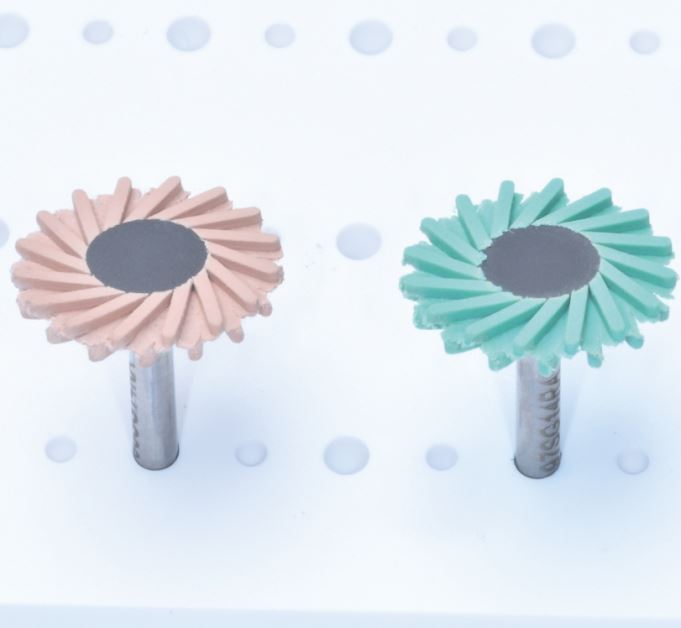
DIATECH ShapeGuard Zirconia Polishing Plus System by Coltene - a two-step system

**Fig. 5:**
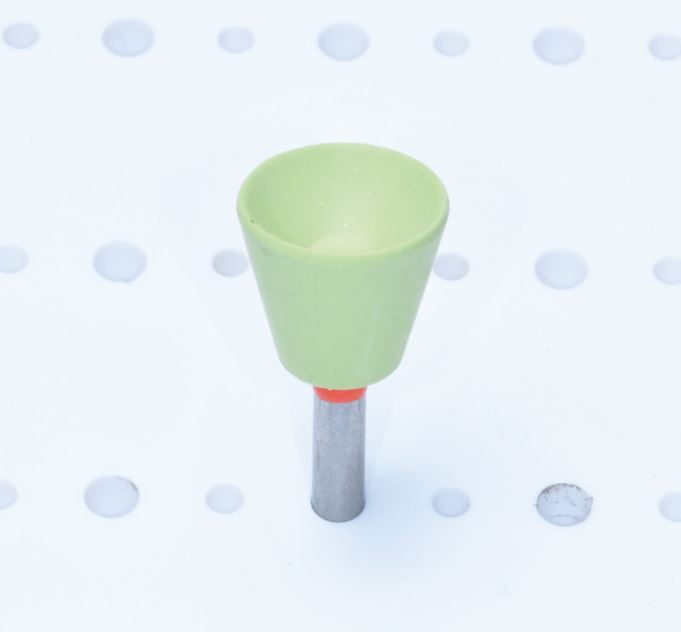
Zirco1 Polishing System by Kenda - a one-step system

**Fig. 6:**
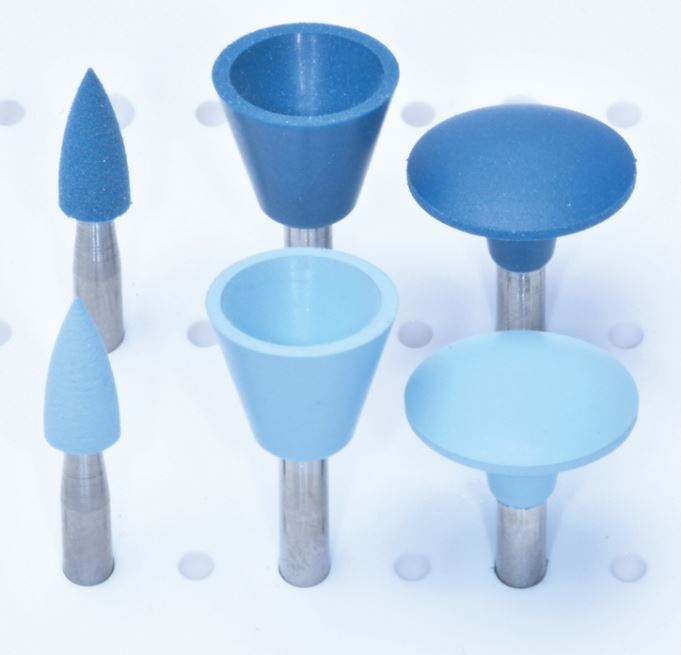
OptraGloss Polishing System by Ivoclar Vivodent - a two-step system

**Fig. 7:**
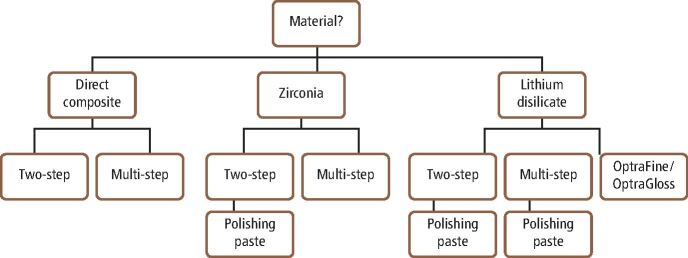
A decision-making flowchart to aid with the choice of polishing system. The process follows the material choice first then the number of steps and finally the potential adjuncts which had showed clinical effectiveness

The use of material-specific polishing systems is effective for chairside polishing of direct and indirect restorative materials. There is promising evidence suggesting that some reduced-steps composite polishing systems are suitable as an alternative to multi-steps systems, producing similar/superior surface roughness and gloss, though this is limited to systems with a minimum of two steps. However, it is important to emphasise that for optimum outcomes, it is essential to follow manufacturers' recommendations for each step, with particular considerations of the handpiece speed, time spent per step and use of adjunct water coolant. Within a clinical setting, there may be a temptation to rush the polishing protocol, but each system must be used effectively with a recommended 60 seconds per step to achieve the most effective results, unless different timings are recommended by the manufacturers. The marketplace for polishing systems is very crowded, with numerous different step systems and materials being considered. There is a lack of classification of polishers and this can lead to confusion regarding what type of system is best. Table 1, Table 2 (see Fig. 4 and Fig. 5) and Table 3 (see Fig. 6) summarise the current commercially available polishing systems for direct and indirect restorative materials which can be used as a navigation guide for clinician. These can be combined with the proposed decision-making flowchart (Fig. 7) to choose the most appropriate system. The only system types included were ones that had evidence of clinically acceptable results within the literature. Some pathways do not include any adjuncts as these achieved appropriate results without them, or already had them incorporated into their system, such as the OptraFine system.

**Table 2:** Table indicating the different zirconia polishing systems available

**Steps**	**Composition**	**Brand name − (abrasive)**	**Manufacturer**
Multi-steps	Rubber points/cups/discs	StarGloss RA − (D)	Edenta
		ZiLMaster − (D)	Shofu
		All Ceramic Intra-Oral Kit* − (?)	Cosmedent
		Twist DIA for Zirconia − (D)	Kuraray
		ZENOSTAR Polishing Set* − (D)	Wieland Dental
		eZr Intra-Oral Adjustment Kit* − (D)	Garrison Dental Solutions
		Luster Intraoral Polishing Kit* − (D)	Meisinger
	Diamond burs, rubber points/cups	LD/ZR Adjustment Kit* − (D)	Komet
Two steps	Rubber points/cups/discs	Luster for Zirconia Adjusting and Polishing Kit − (D)	Meisinger
		EVE Diacera Diamond Rubber − (D)	Eagle Dental
		StarTec − (D)	Edenta
		DIATECH ShapeGuard Zirconia Polishing Plus − (D)	Coltene (Fig. 4)
		Dialite ZR Polishers for Zirconia − (D)	Brasseler
		Jiffy Universal* − (D)	Ultradent
		Set 4622* (D)	Komet
		EZPZ Intra-Oral Zirconia Polishing System Kit − (D)	Maverick Dental
		Zir Gloss Intra-Oral − (D)	Jota
Single steps	Rubber points/cups/discs	Identoflex Diamond Ceramic Polishers − (D)	Kerr
		Zirco1 Polisher − (D)	Kenda (Fig. 5)
Key: D = Diamond ? = Unknown * = Indicates a system used for both zirconia and lithium disilicate

**Table 3:** Table indicating the different lithium disilicate polishing systems available

**Steps**	**Composition**	**Brand name − (Abrasive)**	**Manufacturer**
Multi-steps	Rubber points/cups/discs	CeraGloss − (D)	Edenta
		Ceramisté − (SC)	Shofu
		DIATECH ShapeGuard Ceramic Polishing Plus − (D)	Coltene
		All Ceramic Intra-Oral Kit* − (?)	Cosmedent
		ZENOSTAR Polishing Set* − (D)	Wieland Dental
		eZr Intra-Oral Adjustment Kit* − (D)	Garrison Dental Solutions
		Luster Intraoral Polishing Kit* − (D)	Meisinger
	Rubber points/cups, brushes/paste	OptraFine − (D)	Ivoclar Vivadent
	Diamond burs, rubber points/cups	LD/ZR Adjustment Kit* − (D)	Komet
Two steps	Rubber points/cups/discs	Exa Cerapol − (D)	Edenta
		EVE Diapro Lithium Disilicate Polishers − (D)	Eagle Dental
		Luster for Lithium Disilicate, Intra-Oral Polishing Kit − (D)	Meisinger
		OptraGloss − (D)	Ivoclar Vivadent (Fig. 6)
		Dialite LD Polishers for Lithium Disilicate − (D)	Brasseler
		Jiffy Universal* − (D)	Ultradent
		Set 4622* (D)	Komet
		CeraMaster Assorted - (D)	Shofu
Single steps	Rubber points/cups/discs	Cerapol Plus − (D)	Edenta
		CeraMaster Course − (D)	Shofu
Key: D = Diamond SC = Silicone carbide ? = Unknown * = Indicates a system used for both zirconia and lithium disilicate

## Data Availability

The data referenced within the paper is freely available from PubMed and is included within the reference list.
