# Correction to: Polishing systems for modern aesthetic dental materials: a narrative review

**DOI:** 10.1038/s41415-024-8117-x

**Published:** 2024-11-22

**Authors:** Adil Khan, Nicholas Hodson, Asmaa Altaie

**Affiliations:** 41415302494001 Clinical Teaching Fellow in Restorative Dentistry, Level 6 Worsley Building, Leeds Dental Institute, Clarendon Way, Leeds, LS2 9LU, UK; 41415302494002 Professor/Honorary Consultant in Restorative Dentistry, Level 6 Worsley Building, Leeds Dental Institute, Clarendon Way, Leeds, LS2 9LU, UK; 41415302494003 Senior Clinical Lecturer/Honorary Consultant in Restorative Dentistry, Level 6 Worsley Building, Leeds Dental Institute, Clarendon Way, Leeds, LS2 9LU, UK

Author's correction note:

Review article *Br Dent J* 2024; **237:** 607-613.

When initially published, the author list order was incorrect. The correct order is:

Adil Khan,*^1^ Nicholas Hodson^2^ and Asmaa Altaie^3^

^1^Clinical Teaching Fellow in Restorative Dentistry, Level 6 Worsley Building, Leeds Dental Institute, Clarendon Way, Leeds, LS2 9LU, UK; ^2^Professor/Honorary Consultant in Restorative Dentistry, Level 6 Worsley Building, Leeds Dental Institute, Clarendon Way, Leeds, LS2 9LU, UK; ^3^Senior Clinical Lecturer/Honorary Consultant in Restorative Dentistry, Level 6 Worsley Building, Leeds Dental Institute, Clarendon Way, Leeds, LS2 9LU, UK.

The authors apologise for any inconvenience caused.

